# Suit for Damages for Using Sulphuric Ether

**Published:** 1858-10

**Authors:** 


*Suit for Damages for Using Sulphuric Ether.*—We learn that Dr. W. T. G. Morton, one of the discoverers of the use of ether as an anæsthetic agent, has commenced a suit for damages against Dr. Davis, surgeon of the U. S. Marine Hospital at Chelsea, for an infringement of his patent, laying his damages at $5000. The discovery of anæsthesia may have taken place before the time of some of our readers, who may, therefore, be unfamiliar with the war which was waged between Drs. Jackson and Morton, who both claimed priority in its discovery. This occurred as long ago as 1846, and at that time, a joint patent was taken by Drs. Jackson and Morton. Their course was censured at that time by most medical journals, but most surgeons and dentists made use of it as an anæsthetic agent without leave or license, and had no trouble. It strikes us then with astonishment, that at this late day, Dr. Morton should commence a suit for infringement. If he succeed, what will be the result? Will every hospital and every surgeon or dentist who has been in the habit of using the agent be liable to Dr. Morton for damages? This becomes an exceedingly knotty question, and one upon which we are not qualified to give an opinion. Some would take the stand that a man has no moral right, according to our ideas as regards discoveries which alleviate human suffering, to keep it from the world; while others would consider that the discoverer of so important an agent would be entitled to compensation of a tangible character, and be justified in getting it as he could. It would seem to us to be a matter of doubt whether a medicinal agent, which has been long known, could be patented in this way for a particular mode of use; but we have no opinion, as we before remarked, as to the merits of the case, and merely give to our readers the item of news.

We learn also from a communication signed "W. E. C.," to the Boston Journal, that Dr. Morton had made a proposition to a "medical gentleman, high in public office, to enter into an amicable suit against him, in order to establish a prestige for him (Morton) under which he could more successfully and effectually carry on his operations in Congress. This offer was spurned with a scorn and contempt commensurate with the high honor of the man to whom it was made." The same correspondent also notices an announcement in the daily prints, that the suit against Dr. Davis is an amicable one, and calls upon Dr. Davis for an explicit denial. In justice to Dr. Davis, we publish the following note which appeared from him in the next number of the same journal:

NOTE FROM DR. DAVIS—REPLY TO W. E. C.

United States Marine Hospital, Chelsea, Sept. 18th, 1858.

Messrs. Editors,—I was amazed to find in the Journal of Thursday, Sept. 16th, which came to hand only this morning, a communication, signed W. E. C., containing charges of the gravest nature against my professional character, in connection with the suit brought against me in the Circuit Court of the United States, by W. T. G. Morton, for an alleged infringement of his patent for the use of ether as an anæsthetic. I was not before aware that I had been charged by a public print, or otherwise, with "disgraceful collusion," and I seize the first moment, after it is brought to my knowledge, to deny *in toto*, as utterly groundless and false, not only this charge, but the various insinuations and imputations so wantonly thrown out by W. E. C. If the writer of the communication, or any gentleman of the profession, will call upon me, I will show him, with pleasure, what I have done, "what I am doing, and what I mean to do;" and moreover satisfy him that, thus far, I have acted under the best legal and medical advice that I could command.

Charles A. Davis,
Physician and Superintendent.

Every physician will of course feel a deep interest in the result of this suit, especially as ether is now becoming more generally used than chloroform, particularly in hospitals. We will keep our readers advised of its progress.

---

*Syrup of the Hypophosphates.*—We have lately seen some very elegant forms of these preparations, at the Drug Store of Mr. Wm. H. Peabody, of this city. Their value in tuberculosis has been very much vaunted by high authorities, and without doubt they exert a beneficial effect upon the disease; like all other new remedies for consumption which possess any virtue, they seem to have been overrated. In this connection we would call attention to the advertisement of Mr. Peabody; he is a young man who has had much experience in the compounding of prescriptions, and who devotes his personal attention to this department exclusively; he was for several years in the store of Mr. Mathews of this city, who has long been known to our readers as a most excellent and reliable druggist. We wish him all success, and he well deserves it.

---

*A New Belt Truss.*—We wish to call attention to a communication to the profession, from Dr. H. H. Reynolds, which is in our advertising department. Dr. Reynolds has long been a sufferer from hernia, and has been led to invent this form of truss, which he has found more comfortable and effectual than any other. Since his former advertisement in this Journal, he has made some important improvements, for a detail of which we refer to the advertisement.

